# Cultural imperatives and the ethics of verbal autopsies in rural Ghana

**DOI:** 10.3402/gha.v6i0.18570

**Published:** 2013-09-19

**Authors:** Raymond A. Aborigo, Pascale Allotey, Paulina Tindana, Daniel Azongo, Cornelius Debpuur

**Affiliations:** 1 Navrongo Health Research Centre, Navrongo, Ghana; 2 Global Public Health, MONASH University, Sunway Campus, Selangor, Malaysia; 3 The Ethox Centre, Department of Public Health, University of Oxford, Oxford, United Kingdom

**Keywords:** verbal autopsy, research ethics, bereavement practices, demographic and health surveillance, maternal mortality, institutional review boards, feedback, confidentiality, Ghana

## Abstract

**Background:**

Due to a paucity of statistics from vital registration systems in developing countries, the verbal autopsy (VA) approach has been used to obtain cause-specific mortality data by interviewing lay respondents on the signs and symptoms experienced by the deceased prior to death. In societies where the culture of mourning is adhered to, the use of VA could clash with traditional norms, thus warranting ethical consideration by researchers.

**Objective:**

The study was designed to explore the ethics and cultural context of collecting VA information through a demographic and health surveillance system in the Kassena-Nankana District (KND) of Ghana.

**Study Design:**

Data were collected through qualitative in-depth interviews (IDIs) with four field staff involved in the routine conduct of VAs, four physicians who code VAs, 20 selected respondents to the VA tool, and eight opinion leaders in the KND. The interviews were supplemented with observation by the researchers and with the field notes of field workers. Interviews were audio-recorded, and local language versions transcribed into English. Thematic analysis was performed using QSR NVivo 8 software.

**Results:**

The data indicate that cultural sensitivities in VA procedures at both the individual and family levels need greater consideration not only for ethical reasons but also to ensure the quality of the data. Discussions of some deaths are culturally prohibited and therefore lead to refusal of interviews. Families were also concerned about the confidentiality of information because of the potential of blame for the death. VA teams do not necessarily engage in culturally appropriate bereavement practices such as the presentation of tokens. The desire by families for feedback on the cause of death, which is currently not provided by researchers, was frequently expressed. Finally, no standard exists on the culturally acceptable time interval between death and VA interviews.

**Conclusion:**

Ethical issues need to be given greater consideration in the collection of cause of death data, and this can be achieved through the establishment of processes that allow active engagement with communities, authorities of civil registrations, and Institutional Review Boards to take greater account of local contexts.

Accurate information on overall and cause-specific mortality is essential to prioritize the activities of health systems and to invest scarce public health and medical care resources efficiently (1–3). The infrastructure for the collection of vital statistics, supplemented by medical expertise, is critical to this process. However, in many parts of the world, particularly in low-income countries, this system is compromised by a combination of poor administrative systems, lack of human resource capacity, and nonconformity by citizenry to reporting regulations. Many people die at home, and the cause of death is particularly difficult to determine even if a death is notified. The verbal autopsy (VA) method was developed to address this problem (4–6). Interviews are conducted with relatives of the deceased to obtain details about the circumstances leading to the death, including the signs and symptoms of any illness prior to the death. These data are then assessed to determine a probable cause of death based on techniques ranging from expert physician decision making to mathematical algorithms (7–9).

The World Health Organization recommends the use of VAs in order to improve the comparability of causes of death from systems without medical certification of deaths (6). For countries with deficient vital records, MEASURE Evaluation has developed Sample Vital Registration with Verbal Autopsy (SAVVY) for strengthening vital events monitoring and measurement, including causes of death (10). In the context of research, Demographic Surveillance Systems (DSSs) have adopted VAs for investigating deaths (11). In order to enable cross-country comparisons, significant efforts have been made in standardizing the protocols for VA data collection and in enhancing the validity of cause of death determination (12–14). However, in standardizing the protocols, the ethics of the procedure has not been given adequate attention. There has been less methodological development to address issues related to protecting VA participants and maximizing benefits to communities than there are to validate and standardize the method. According to Chandramohan et al., VAs can aggravate the grief and emotional distress of families and communities (15).

Other procedures related to revisiting death narratives such as vital registrations are also likely to cause some degree of distress and may account in part for levels of civil registrations. However, civil registration is a critical function of the state, not only to support national policy but also to trigger legal processes relating to the state of the deceased. There is no doubt that civil registration processes need to be improved, not only to acknowledge the distress of bereaved relatives but also in terms of the infrastructure, accessibility, and quality of the records (6).

The collection of data using the VA procedure provides an opportunity to explore and address the role of distress in reporting deaths and determining the cause of death. Arguably, distress may affect the integrity of the data collected and can raise significant ethical concerns for both researchers and civil registration authorities who visit families for death narratives.

The aim of this qualitative study was to explore the cultural context of VA data collection through a demographic surveillance system in Ghana. The specific objectives were:

- To describe bereavement practices in the district;
- To investigate the experiences of field staff involved in VA data collection; and
- To identify ethical issues in VA data collection from the perspectives of community members, respondents of VAs, and VA teams.

## Methods

*The research site*: The study was conducted in the Kassena-Nankana District (KND), which has a population of about 150,000 with three related but distinct ethnic groups. The district is one of the poorest in Ghana with subsistence farming being the mainstay of the people. The KND is largely patriarchal with strict gender roles. Generally, caring for sick people is the role of women, and only rarely do men take up such responsibilities. A study by Tindana et al. in the research setting found that their sample of parents of children who had previous research experience were all women (16).

The district is home to the Navrongo Health Research Centre (NHRC), which runs the Navrongo Health and Demographic Surveillance System (NHDSS) as a continuous vital registration system that monitors the health and population dynamics of the inhabitants of the district. The surveillance covers an area of 1,675 km^2^, with 30,500 households. The entire population is followed on a 120-day cycle to monitor vital events, including births, deaths, and migrations. The system also registers and monitors pregnancies and their outcomes to enhance the reporting of births, as well as neonatal and maternal deaths. Most deaths that occur in the surveillance site are identified by community-based data collectors and followed up by trained field staff who collect data for VAs. VA data are collected using a structured questionnaire standardized by researchers within the International Network of field sites with continuous Demographic Evaluation of Populations and Their Health (INDEPTH) (17). The data are independently coded by three physicians to assign the cause of death using the *International Classification of Disease*, 10th revision (ICD-10) (18). Where at least two of the physicians agree on a diagnosis, it is accepted as the cause of death. Where there are disagreements, the case is set aside for further review by a third physician. In case of further disagreement by the third physician, the case is coded as undetermined. A detailed description of the NHDSS has been reported elsewhere (19–21).

At the commencement of surveillance activities (about 21 years ago), the NHRC did not have an ethics committee and so the research tools, including the VA tools, were not taken through any formal ethics review process. Approval for surveillance activities was obtained from the Ministry of Health. Currently, the NHRC has an Institutional Review Board (IRB) with a Federal Wide Assurance (FWA00000250), and both the VA and other surveillance data collection tools have been reviewed and approved by the Board (NHRCIRB115).

Following the regulatory approvals, intense community engagement activities were pursued by the NHRC before initiating data collection in the field (22). Community meetings were held with people and opinion leaders in various communities, and the objectives and procedures involved in surveillance activities were explained to them in order to obtain their support. Verbal consent was sought at the individual level, and the content of the form included the purpose of the study, procedures involved, risks and benefits of participation, opportunity to withdraw, confidentiality of the data, and contact persons in case individuals needed further clarification.

### Study design

In-depth interviews (IDIs) were conducted with key informants, and data were triangulated with observation and field notes. The data were collected in August 2010.

Two graduate-level research assistants who were fluent in the local languages of the study area and had about three years of experience in moderating focus group discussions (FGDs) and conducting IDIs within the research setting were employed to carry out the interviews. They were trained on the research protocol and the data collection tools and consent procedures for a period of two weeks. The data collection tools were pilot tested in the field, and all necessary amendments made before they were deployed to the field. All the community-level interviews were conducted in the local languages (Kasem and Nankani), while those with the field staff and coders were done in English.

### Sample

For the purposes of data collection, the NHDSS has divided the district into five zones – the East, West, North, South, and Central zones. The East and South zones are Nankanis, while the North and West zones are Kassenas. The Central zone is cosmopolitan and was therefore not included in the sampling frame. One of the zones was randomly selected to represent each of the ethnic groups – the East for the Nankanis and the North for the Kassenas. Within the zones are clusters that are made up of a maximum of 99 compounds. One cluster was randomly selected from each zone. The community key informant (CKI), whose catchment area included the selected cluster, was invited for an in-depth interview – two in all.

In establishing the NHDSS, a team of CKIs were engaged by the centre to record all pregnancies, births, and deaths that occur in their localities to complement routine interviewer coverage (19–21). Community leaders select CKIs based on their honesty, literacy, knowledge of the traditions of the community, and willingness to work for a token fee. The CKIs assisted the research team in identifying two chiefs, two ‘landlords’, and two district assembly members for IDIs. Chiefs and landlords are the repositories of traditional knowledge, while assembly members may have both traditional and political knowledge of their communities. CKIs, chiefs, landlords and assembly members are opinion leaders. The determination of who was invited to participate in the study was left to the discretion of the CKIs, who were guided by their traditional knowledge of the community and a minimum criterion to select individuals who could provide cultural knowledge on bereavement as it relates to the VA process. The researchers could only confirm the appropriateness of the participants during the interviews.

The NHDSS has four core field staff responsible for VA data collection. All four were included in the list of respondents to explore their understanding of the local culture and their experiences with VA data collection. Other key informants included four out of the five expert coders (the fifth was not available) to explore their views about potential ethical issues that they may have identified in their review and utilization of VA data. Coders are research clinicians who understand and appreciate the VA process, and therefore their views were relevant in shaping the data collection process. Limitations in the data that they use in making the cause of death determination could lead to a significant number of cases being classified as undetermined, as identified by Allotey and Reidpath (23).

Finally, the 10 most recent (within two weeks of the study) community members to have granted VA interviews were purposively selected as community respondents for the study. Ten additional interviews conducted to make 20 when an initial review of the data revealed further themes to be explored. Two weeks was considered short enough to reduce recall bias so that respondents could adequately share their experiences during their encounter with the VA team. Unsurprisingly, all the community leaders identified were male, and family members interviewed about VAs were female.

### Data management and analysis

All the interviews were tape-recorded and transcribed in English, and thematic analysis was performed using QSR NVivo 8. Two researchers coded the interviews independently. Interpretation of the results incorporated the varied views of the research participants. Verbatim quotes from each of the groups are used to elucidate the issues discussed in this article.

### Ethical considerations

The study was approved by the NHRC IRB (NHRCIRB093). Informed consent was obtained from participants in their preferred language after they were told the purpose of the study, procedures involved, risks and benefits of participation, right to withdraw or refuse to participate during the interviews, procedures to ensure that participants are not identified in relation to their study information and contact people in case interviewees needed further clarification.

## Results

### Bereavement and mourning

Deaths in the KND are marked by elaborate funerals consisting of traditional performances and rituals with a focus on mourning the loss. Mourners and sympathizers include close and distant relatives, prominent people in the community, and friends of the deceased and those of family members. The elaborateness depends on family wealth and social status. Regardless of the wealth of the family, however, there are traditional expectations that all visitors to homes where a death has occurred contribute something in cash or in kind to show sympathy and to acknowledge the real loss in resources to the family as a consequence of the death. In real terms, these in-kind contributions may be drinks, kola nuts, and livestock. This contribution is necessary regardless of the age or role of the deceased. However, the value of the contribution varies depending on the social status of the deceased, as the following shows:

Let's take it that it's a chief who died, you have to go to an elderly person who is always with the chief and can give you the information. So going to see those people, you must send kola-nuts and drinks to greet them before you ask them. (VA respondent)

As with most traditional practices, there have been some changes with increasing development of the rural areas. The duration of mourning, for instance, used to be predictable and related to the time of burial and the age of the deceased. However, with the advent of refrigeration this period is harder to determine, as illustrated by the excerpt below.

It depends on who has died and whether the family has money or not. Nowadays, we have fridges and those who can afford it can put their dead people there while they prepare for the funeral but that is for the old people who die. They put the dead person in the fridge to prepare for the funeral, to inform in-laws, friends, relatives and people who live outside the village because they all have to put resources together for the funeral. Sometimes it can take a year before the funeral but that is if you have [wealth]. If you have money you can give those who come to mourn with you beer and take-away (pre-packed lunch); if not, you just give them water or pito [locally brewed alcohol made from sorghum]. (Opinion leader, assembly member)

### Engaging with relatives of the deceased

Although the bereavement and mourning practices were well known by the field staff, they did not always follow these procedures during visits for VA data collection. As part of the standard procedure of the research centre, and in adherence to ethics requirements, researchers and field staff from the research centre are not authorized to offer any gratuities to avoid any perception of coercion to participate in research. The engagement with bereaved families is limited to offering sympathies and obtaining verbal consent before carrying out the interview. The limited engagement had been noted by some of the respondents.

That one, I think that if the Centre [Navrongo Health Research Centre] can always give us something that we can give to them after interviewing just to say thank you as a way of sympathizing with them because in our local homes when you go to greet someone in the house you have to show some level of respect or concern. When the Centre is able to do this, the people will be ready to give the correct information …. They [Navrongo Health Research Centre] say they don't have money to do that and that they don't want the people to be forced to take part because of that. (Data collector)

### Emotional distress

Emotional distress was described in different terms by participants. Community leaders spoke about the importance of both nuclear and extended families and the extent of grief depending on the relationship and the role of the deceased. Data collectors spoke about encountering severe distress in families where the loss was of an only child or where both mother and infant were deceased. They reported several instances where data collectors were denied interviews by relatives of the deceased.

Mostly they refuse to do [the interview] because she has lost her only child and your interview has nothing to do with its come back. (Data collector)

Similarly, respondents who were in a close relationship with the deceased and those for whom the deceased was the sole source of income also expressed distress and reluctance to engage in VAs.

Because I lost my old lady and she came to remind me of her again and when she asked me the question and I was narrating to her, it was disturbing me in the heart and I wanted to stop her from asking me those questions. (VA Respondent)

The nature of the death and therefore the core subject of the VA were also significant in the severity of distress caused by the VA interview. Specific circumstances that caused more distress included death from a short illness or a road traffic accident because these were sudden and unexpected. The grief process for such deaths was therefore described as qualitatively different.

For the road traffic accidents when you get to that point, it is always pathetic because many are always crying. When you ask for the history, it is that part that they start to shed tears. For instance, when they narrate it and you start to write, before you raise your head, they have already started crying. (Data collector)

The VA team reported that efforts have been made over the years to be sensitive to cultural issues by extending the period between the death and interview, but the strategy has not been very successful as some women still break down in tears during the interviews regardless of the length of time following the death.

So there may be problems because often the concerns are that their relatives have died and people are mourning and you go and remind them again. It is something which is a bit of a problem at times, so what they have done over the years is to be very sensitive to those cultural issues and give some time to the people to get over the death before you approach them to talk about it and even [then] I am told when you are talking about the child death particularly, the mothers break down and cry. (Coder)

### Timing of VA interviews

Field staff reported that there are no specific instructions from the research perspective on how soon after death the VA should be conducted. The only instruction is that it should be recent enough to avoid recall bias. This means technically that interviews can be conducted as soon as the field staff is notified of a death, or much later if the death is not identified until the census round. There was no cultural guidance either; that is, there are no taboos on when the events leading up to the death could be spoken about. However, there were instances where mourning and associated rituals were carried out over a protracted period of time. Data collectors either had to wait out this period or risk offending cultural sensitivities by ignoring them.

For neonate, infant, and child deaths all are seen as children – so after the burial it can take two weeks before we go to conduct the interviews because if you go immediately they may still be mourning and they wouldn't be able to give you the right information. But for maternal death that is an adult, a week or two is okay. The ‘spirit child’ usually takes longer than the rest because they don't even want to admit that such a person was killed because they see it to be a disgrace to their families since everyone has the perception that a ‘spirit child’ is capable of destroying a whole house – so they have to eliminate it before it starts to kill people. (Data collector)

Furthermore, the availability of functioning morgues has resulted in protracted funeral arrangements for ethnic or religious groups that do not have strict requirements for how soon after death the burial needs to occur. Although generally it takes between one and three months before VAs are conducted, sometimes VAs are carried out within a week of the death.

So at times it takes about a month before we go to interview the person, and other times it can take only one week. (Data collector)

### Feedback on cause of death

Within traditional systems, knowing the cause of death is important; it holds meaning for the bereaved, and it is important to guide the spirit of the deceased. The ‘spiritual’ cause is often determined by soothsayers, traditional practitioners, or religious people. Such consultations are usually commissioned by compound heads. In order to avoid confusion in the family, the outcomes of such consultations are often kept secret and dealt with according to traditionally laid-down protocols.

There are bad spirits in this world. Nobody dies out of nothing but there are certain deaths that you can never know their cause but the compound head who doesn't sleep at night will know. They can go to a ‘vuru’ [soothsayer] to know all what happened but when they find out they keep it secret. They don't let it out to people else it will cause confusion in the house. Only the elders get to know. If you people can tell us why the person died, maybe we will not go out looking for it again to cause problems. (Opinion leader, landlord)

When researchers seek consent from families to determine a clinical cause of death, the expectation given is that the data collected will lead to further information to provide some meaning to the loss. However, it is not standard practice for researchers to return to individuals or families to provide them with this information once it has been processed. The reason given is that coding is based on expert decision making rather than the exact cause of death. Feedback has traditionally been at the community level, where the broad causes of death are explained to community members.

That is not our business. In conducting VAs, our business is to look for the cause of death, period. At a later time we can talk of interventions not to individuals or families because at the end of the year usually there are reports that are generated and there are disseminations that are carried out in the communities on all studies and these things are captured there as causes of death. What is probably killing the people or what are the diseases that are killing the people. But as [of] now, we do not go back to tell the families the causes of their relatives’ deaths. Our business is to ask them about the causes of the deaths and how your relative died and we will use our scientific methods together with your narrative and assign a particular cause to it. Some cases we can't confirm it exactly so we actually have no business to go back and say 100% this is the actual causes of a death that killed Mr A or Mr B. (Coder)

Lack of feedback at the family level was raised by all families and communities. Some specific questions were raised where there was a possibility that it was a preventable illness.

If they tell you and you know, you can always prevent yourself from these diseases so that in future, such a thing will not happen to you because you might not know but those who came and conducted the interview will know so when they give you the feedback it will be better. (VA respondent)

### Confidentiality of VA data

Respondents highlighted a number of concerns about confidentiality relating to the process of data collection, the content of the information that was collected, and ultimately what the cause of death may turn out to be. One particular concern was the one-to-one interview ideal which reflect Western norms of medical information given but may not necessarily apply in collectivist societies such as that of the Kassena-Nankana District. For instance, when data collectors went to collect data within the compound, they could not control who was present during the interview – particularly since a death was the sort of event that brought family members together to support each other. Not only was it difficult to guarantee confidentiality within the household, but also there were implications, both positive and negative, for the quality of the data collected.

If a man dies and you are interviewing his wife and the compound head is around you cannot sack him. He also sits and listens because you have already explained to him why you came. So where the woman cannot provide certain information that you need, the compound head can chip in and say can you remember this or that happened? (Data collector)

Concerns about confidentiality were also raised about deaths that were potentially as a result of stigmatizing diseases.

In this community, if people see you growing lean, they will start pointing hands and saying things, so if you people let the people know that what they were talking of is true, it will bring problems to us [the family]. (VA respondent)

The coders cautioned against giving out information that had the potential to stigmatize communities and advised VA teams to be careful not to confuse their role as researchers with that of criminal investigators. As researchers, VA teams are bound by certain ethics, which include protecting their participants.

The problem may arise when you give out information that has to do with, excuse me to say, a stigmatized disease, for instance like the cause of death being HIV/AIDS or a narrative that suggests a death was due to HIV/AIDS. These are where ethical issues may arise where repercussions could befall the family members of the deceased. These are sensitive or medically sensitive information which we do not have a degree of control over because we are not in a hospital setting. Also, under the law, we can be called to bear witness in court if it is a criminal case and this can be tricky. We wouldn't know what to do. (Coder)

### Regard for cultural prohibitions

In the Kassena-Nankana society, it is considered a taboo to mourn or discuss the death of a *chuchuru* (spirit child)^1^ in the family. Bad omens such as the death of family members that are often associated with a ‘spirit child’ make it culturally inappropriate to probe mothers for information about the circumstances of the death. According to the community members, talking about a ‘spirit child’ exposes the family to the risk of having the child reborn with the same characteristics, behaviours, and consequences. VA procedures, however, require that data collectors visit close relatives of the ‘spirit child’ to enquire about the circumstances surrounding the child's death. Though the VA team is aware that asking people to talk about a ‘spirit child’ violates traditional norms and could jeopardise community relationships that support cohort maintenance, they are obliged by the VA process to still visit such families.

[I]t can take so many years and if the house members don't realize it early, it [spirit child] can grow old to about 10 years or until ‘we napiiri o, o pa debam’ [God is able to identify it for us]. Because in most cases it can kill its own relatives until such a time that they realize it is not a good thing, then they tell the ‘kwo-bia’ [tribal playing mates] because if you have a ‘chuchuru’ [spirit child] in your house and you even go out to consult with the ‘vura’ [soothsayers], it [spirit child] will quickly go and block all the ways they can use to know that it is a chuchuru such that you cannot identify it. But, the tribal play mates can ‘fogibayiga’ [prepare themselves spiritually, and] identify and kill it. (Opinion leader, chief)

Like I have said, if a chuchuru dies and the chuchuru undertakers come to carry it away, nobody comes to greet the funeral because the perception is that if people come to greet, it will come back again. So they go to throw it away and never to come back at all. (Opinion leader, CKI)

Data collectors often find out about a ‘spirit child’ through the use of euphemisms like ‘the child was thrown into the bush’. Community members believe that some ‘spirit children’ are able to hide themselves from soothsayers and are not discovered until the child is up to 10 years old. If the family is ‘fortunate’, then God will help them identify the spirit child, but generally, it is believed that only the *kwo-bia* (tribal playmates), who have the spiritual powers, can identify them.

You know nobody wants to have a spirit child in his home because it is a very destructive being. So the moment you realise that it is a spirit child we get some particular people to kill and bury it in the bush. (VA respondent)

Also, in the Kassena-Nankana tradition, it is prohibited to discuss the death of a traditional authority such as a chief or a landlord prior to the formal announcement of the death by the family. Funerals of this category of people are elaborate and logistically demanding. Families therefore require adequate time to gather resources and to inform all who need to be present at the funeral.

In our ‘Kassena-chonga’ [Kassena tradition] it can take about one or two months before people get to know that the chief is no more. Nobody talks about it until it is announced and others get to know when they see certain things happening around the house. The landlords too, they hide and bury them. For instance, if a landlord dies anywhere, they will secretly come and inform the chief that one of his subordinates is no more. So if there is a problem and he was supposed to be around him to solve it you will know because they will send a representative. They secretly bury him and get the necessary people to perform the necessary rites before they let people hear about it because it also pulls crowd. (Opinion leader, chief)

## Discussion

This study reveals the cultural sensitivities in VA procedures at the individual, family, and community levels that need greater consideration not only for ethical reasons but also to ensure the quality of VA data. Discussions of some deaths are culturally prohibited, and confidentiality is needed for cohort maintenance. VA teams are called upon to engage in culturally appropriate bereavement practices such as the presentation of tokens and to provide feedback on cause of death. The need for VA teams to consult with communities for guidance on the most appropriate time interval between death and VA interviews was emphasized. A summary of the study findings are contained in Table 1.

**Table 1:** Key findings

Traditional expectations exist for all visitors to homes where a death has occurred which include the contribution of something in cash or in-kind to sympathize and acknowledge the real loss in resources to the family. VA teams do not offer gratuities to families of the deceased based on perceived coercion to participate in the VA process. Emotional distress is influenced by the relationship between the respondent and the deceased, the role of the deceased in the family, the lost of an only child, the lost of both mother and infant and the nature of the death. VA teams have no standard protocols for the timing of VA interviews. This includes how long after death and at what stage of the funeral the interview should be conducted. It is not standard practice for VA teams to return to individuals or families to provide feedback on cause of death but community members are expressing interest in receiving feedback. All carers for the deceased are usually brought together for VA interviews and this has implications for confidentiality and the quality of VA data. Discussion of some deaths are culturally prohibited and therefore lead to refusal of interviews.

DSSs, established primarily for longitudinal research and used for community-based intervention trials, are subject to ethics principles. Critical for the success and, arguably, the ethical conduct of the research is the need to establish and regularly negotiate a relationship with the community. A good relationship with the community is important to mitigate the pervasiveness of ongoing data collection. Essentially, this means that DSS research teams need to take on community engagement activities that complement data collection. Such activities will go beyond the community engagement process outlined by Tindana et al. (22) to include adherence to local customs, such as funeral rituals that require gift giving that should not necessarily be assessed under the ethics criteria of incentives or coercion (24–27). Indeed, any use of VA, including civil registrations, should be guided by basic principles of collaboration (28) with communities and should seek as a final outcome the empowerment of communities to ensure that the process of accessing mortality data does not impose practices that conflict with traditions. This can be achieved through communication and meaningful consultation with communities to establish partnerships during the design of VA surveys and throughout the data collection process.

The involvement of CKIs or community advisory committees who are knowledgeable in the local customs and culture of the people would further support VA teams regarding the extent to which traditional protocols need to be observed, particularly for families of the deceased. This could include timing of the interviews, gifts to be presented to the bereaved family, and traditional expectations for specific deaths. In some cases, visits to sympathise with the bereaved family prior to the day of the interview may be recommended to learn the best approach to conducting the interviews and also to prepare relatives of the deceased for the impending interview. Considering the huge number of deaths that civil registrations using VA approaches deal with, it may be impossible for such systems to go beyond sympathizing with relatives of the deceased in their engagement process. However, professionals engaged in the process should be capable of preparing individuals or families psychologically for the impending VA interview by sympathizing with them appropriately.

Although intense community engagement activities were carried out at the start of surveillance activities in the KND in 1992 (19, 20, 22), individuals participating in VA interviews may still require information with regard to VA procedures and the overall objective of the activities. This is often conveyed to respondents during the informed consent process, which has traditionally been verbal. Data collectors are not required to document the consent of participants before carrying out their interviews. However, considering that the VA process is beyond minimal risk, there has been a call for written informed consent for participants in VAs (15). In settings where VA is used for civil registrations, individuals and families will be required by law to participate in VA interviews, but this does not absolve the professionals involved in the process from providing them with information on VAs and the objectives of the procedure in a sensitive manner.

The ethical principle of respect for persons, which acknowledges the right of the individual to choose to either participate in a research activity or not, is necessary in the process of enrolling people into VAs. It has been argued that there is total cooperation of inhabitants in surveillance communities, which ‘can threaten the ability of individuals or households to make truly autonomous opt-in decisions’ (29). This is because a decision by individuals and households within surveillance communities, or under compulsory registrations, to opt out has been made almost impossible by their location and by law. Recounting events surrounding the death of a close relative could bring about severe emotional distress (15). In research settings, this demands that the individual is given the opportunity to make an informed decision based on a culturally appropriate consent process. This includes the language used in seeking consent, the feasibility of using written consent, and the possibility of using other approved approaches such as pictographs and ethnographic methods.

Also, Chandramohan et al. (15) have indicated that some field staff on VAs come from surveillance communities, and therefore confidentiality of information cannot be guaranteed. Due to linguistic barriers and the cultural sensitivity of death enquiries, the use of such data collectors may be inevitable. The situation is even more complex when, in order not to compromise the quality of the data collected, all family members who cared for the deceased are brought together to provide information during the interviews. Although this collective provision of information could threaten confidentiality and potentially stigmatize families and communities, it does offer an opportunity for cross verification and validation of the VA data. It is unlikely that the collectivist societal structure that exists in many countries where VAs are performed can be subjected to change and so it may be more feasible and appropriate to consider how the VA mechanism operates effectively within these contexts. One option is to consider separate interviews with all carers for the deceased but the onus lies with VA teams and civil registrars using VA to set up internal structures to protect participants.

Individuals and families who suffer a significant loss may require counselling to calm down their feelings, thoughts, and experiences prior to mortality enquiries. In this regard, it has been suggested that VA data collectors be adequately equipped with counselling skills (15). However, before providing such services, individuals engaged in mortality enquiries will need to understand the bereavement culture of the communities they work in, the different models of mourning, and the different factors that may affect the grief response of individuals and families. Factors that were reported to significantly affect the grief response included the relationship between the respondent and the deceased, the role of the deceased in the family, the loss of an only child, the loss of both mother and infant, and the nature of the death. These factors are similar to those found by Chandramohan et al. (15). For VAs in both research and civil registrations using active methods of data collection, it is important that the counselling approach adopted is designed in collaboration with the community to ensure its relevance. As demonstrated from the results, data on ‘spirit child’ deaths, for instance, would require critical negotiation to facilitate disclosure by families. Clearly, VA staff in both research and civil registrations would have to be equipped with appropriate counselling skills as part of their professional training.

The culture of mourning in traditional African society usually takes a long time to complete (30). The proliferation of functioning morgues has further compounded this problem. This demands that data collectors allow enough time for the family to mourn the deceased before contacting them for VA interviews. The appropriateness of this lies in the suggestion that recall bias is not a problem in recounting tragic events such as death (31, 32). General recommendations regarding the length of time between death and VA interviews range from three months (33) to two years (31, 34). However, beyond the issue of recall bias, researchers and state registration authorities may need to explore more systematically what the appropriate cultural compromise would be for specific deaths and provide the needed guidance to field staff as they endeavour to collect VA data.

In countries where Islam is dominant, like Malaysia, there are cultural imperatives for burials to occur within 24 hours of a death and religious beliefs that preclude post-mortem examinations, which are often perceived as mutilation of the body. Indeed, in such settings, VA approaches may be preferred for cause of death determination. Similarly, within the context of many African cultures, for instance, death is considered inevitable and the loss is often mitigated by the possibility of reincarnation (35). Within several African cultures, the belief is held that the discarnate soul is born again in another body a number of times. The re-embodiment is dependent on the type of funeral rites that are performed, particularly to protect against the reincarnation of social outcasts, public miscreants, lepers, and witches (36). These beliefs are powerfully illustrated in rituals following the death of children in the KND believed to be ‘spirit children’ or *chuchuru*. The belief in ‘spirit children’ is closely bound to maternal and perinatal morbidity and mortality (23, 37), and it is culturally inappropriate to discuss the death of a ‘spirit child’ without provoking traditional sensitivities. The belief system is considered important enough to override any public health requirement to determine cause of death. These belief systems clearly extend to various degrees across many cultures, and the VA protocol and other requirements for ascertaining cause of death need to embed approaches to managing these belief systems within the process of data collection.

Individuals contribute information towards the determination of cause of death, but these individuals do not benefit directly from the VA process. As advanced by the Belmont report, an injustice occurs when some benefit to which a person is entitled is denied without good reason or when some burden is imposed unduly (38). Individuals can benefit from feedback on the probable cause of death of their relative if such information can help either the individual or family take precautions against an impending health crisis. Fottrell and Byass (39) have argued for the use of mortality measures to detect humanitarian crises for public health interventions at the population level. This article extends the discussion to crisis at the individual and family levels. From the study, respondents have often expressed the desire to know the cause of death of their relatives during death narratives, but this request has not been met by VA teams. The arguments offered in the data for not providing such feedback is that cause of death based on VA data does not have the full confidence of physicians. There is, however, no fundamental reason why a physician's opinion based on a VA is different from feedback from a physician issuing a death certificate. Feedback is especially crucial in the case of communicable diseases in order to avoid a health crisis for the family. One could argue that if data from VAs are reliable to inform priorities in health interventions, then such data could be useful to the spouse of an individual who died from HIV/AIDs. Consequently, even though the results of VAs may not have the full confidence of physicians, as purported in the data, they may form the basis for further investigations by the individual or family. The process of feeding back cause of death may risk stigmatizing individuals and families and therefore care must be taken to ensure that only individuals who can benefit from the information receive it. For state registrations systems, feedback to individuals or families may not be feasible due to the huge populations involved but may be a necessary part of the VA process in the research context.

Where individuals opt for feedback on the cause of death of their relative, the surveillance system should be able to provide ancillary care for any investigation that leads to the discovery of the suspected illness in the respondent. Ancillary care is that which is not required to make a study scientifically valid, to ensure a trial's safety, or to redress research injuries (40). Practical steps have been outlined to guide researchers in providing ancillary care (41). According to Carrel and Rennie, the principle of ancillary care is commonly misunderstood to apply to only clinical trials, but participants in routine surveillance systems have the right to such care (29).

### Study limitations

Detailed description of the mourning culture for specific deaths and its effect on distress was not studied as they were beyond the scope of this article. A longitudinal study design will be required to study these relations.

Also, the selection of our sample was based on time to interview and not necessarily the cause of death. We are therefore unable to tell what the impact on our results would have been if our respondents were selected based on the cause of death.

Finally, due to the design of the study, we are unable to give quantitative measurements to the different levels of distress associated with the factors that were reported to influence it.

## Conclusions

With increasing focus on improving the capacity of developing countries to undertake research for health, the establishment of IRBs for ethical research has been given priority (42). This is evident in the increase in funding and other types of support available to enhance the ethics review process in research. In considering the ethical merits of research protocols, IRBs are guided by international guidelines and principles from key documents such as the Helsinki Declaration and the Belmont Report (38, 43). There is, however, no formal procedure for considering the ethics of other methods involving death registrations, such as compulsory civil registrations. That notwithstanding, the principles guiding research studies are almost universally accepted. Although they arise from a particular historical and cultural context and were conceived primarily for the purpose of monitoring clinical and experimental research with human subjects, the principles can be adopted with a commensurate critical analysis of the type of investigations and the cultural context in which the investigations are being carried out.

## Footnotes

1 A “spirit child” is a child born with physical abnormalities such as teeth at birth or whose birth is followed by a series of mysterious events such as incurable diseases or death of family members.
